# Identification of Common Bacterial Pathogens Causing Meningitis in Culture-Negative Cerebrospinal Fluid Samples Using Real-Time Polymerase Chain Reaction

**DOI:** 10.1155/2016/4197187

**Published:** 2016-08-01

**Authors:** Walaa Shawky Khater, Safia Hamed Elabd

**Affiliations:** Medical Microbiology and Immunology, Faculty of Medicine, Ain Shams University, Cairo 11566, Egypt

## Abstract

*Background*. Meningitis is a serious communicable disease with high morbidity and mortality rates. It is an endemic disease in Egypt caused mainly by* Streptococcus pneumoniae*,* Neisseria meningitidis*, and* Haemophilus influenzae*. In some settings, bacterial meningitis is documented depending mainly on positive cerebrospinal fluid (CSF) culture results or CSF positive latex agglutination test, missing the important role of prior antimicrobial intake which can yield negative culture and latex agglutination test results. This study aimed to utilize molecular technology in order to diagnose bacterial meningitis in culture-negative CSF samples.* Materials and Methods*. Forty culture-negative CSF samples from suspected cases of bacterial meningitis were examined by real-time polymerase chain reaction (real-time PCR) for the presence of* lytA*,* bexA*, and* ctrA* genes specific for* Streptococcus pneumoniae*,* Haemophilus influenzae*, and* Neisseria meningitidis*, respectively.* Results*. Positive real-time PCR results for* Streptococcus pneumoniae* were detected in 36 (90%) of culture-negative CSF samples while no positive results for* Haemophilus influenzae* or* Neisseria meningitidis* were detected. Four (10%) samples were negative by real-time PCR for all tested organisms.* Conclusion*. The use of molecular techniques as real-time PCR can provide a valuable addition to the proportion of diagnosed cases of bacterial meningitis especially in settings with high rates of culture-negative results.

## 1. Introduction

Bacterial meningitis is one of the serious communicable diseases, associated with substantial morbidity and mortality rates [1]. About 10 to 20% of survivors develop disabling neurologic complications [2] mandating the prompt diagnosis, treatment, and prevention. The incidence of meningitis is usually high in developing countries, with poor-socioeconomic status [3], and it is in fact reported endemic in Egypt [4].* Streptococcus pneumoniae*,* Neisseria meningitidis*, and* Haemophilus influenzae *type b account for most of the world's documented cases of community-acquired bacterial meningitis [2–6]. However, over the past decade, changes in the epidemiology of the disease regarding the distribution of the causative agents and patients' age group have been noticed in different geographical areas, owing to the implementation of different immunization strategies using conjugate polysaccharide vaccines [1, 3, 7, 8].

Different methods exist for the diagnosis of bacterial meningitis, of which cerebrospinal fluid (CSF) culture is still considered the “gold standard” [9, 10]. However, culture techniques in some settings lack sensitivity particularly when the patient is pretreated with antimicrobials; added to that, the disadvantage of the turnaround time till results becomes available [9, 11–13]. Gram-stained smears of the CSF samples can provide a rapid preliminary tool for diagnosis in 60–90% of patients which correlates with the concentration of bacteria in the CSF samples [9]. However, sensitivities of CSF Gram staining vary considerably for different microorganisms [3]. Some studies have also reported a low sensitivity of direct antigen detection assays as latex agglutination test, especially in pretreated patients with antibiotics before lumbar puncture [14–16].

Accordingly, molecular methods have been proposed to fill in these gaps, as rapid and accurate methods especially in culture-negative situations [6, 10, 17–19].

This study aimed to diagnose bacterial meningitis caused by* Neisseria meningitidis*,* Streptococcus pneumoniae*, and* Haemophilus influenzae *type b (Hib) in culture-negative CSF samples by the aid of real-time PCR.

## 2. Materials and Methods

This study was performed during the period from December 2014 to March 2015 and was approved by the Ethics Committee of Faculty of Medicine, Ain Shams University, Cairo, Egypt, and in accordance with the ethical guidelines of the Declaration of Helsinki, 1975.

The study comprised a total of 40 CSF samples recovered from adult patients, admitted to Abbasseya Fever Hospital, presenting with clinical picture and abnormal CSF cellular and chemistry results suggestive of bacterial meningitis.

Clinical criteria for inclusion of patients were fever, headache, vomiting, photophobia, and irritability (symptoms of meningeal irritation) along with neck rigidity, Kernig sign, Brudzinski sign, altered conscious level, seizures, and focal neurological signs (signs of meningeal irritation) [4].

The diagnostic laboratory criteria for bacterial meningitis included the following: glucose concentration less than 40 mg/L, protein concentration more than 50 mg/dL, a white cell count more than 100 cells per mm^3^, and neutrophil percentage more than 50% [20].

The collected CSF samples were centrifuged at 10.000 rpm for 10 min and the supernatant was examined for abnormalities in WBCs, protein, and glucose. Ten microliters of the sediment was inoculated onto sheep blood agar and chocolate agar and the rest was aliquoted and stored at −70°C. Bacterial growth was observed after overnight incubation of the agar plates at 37°C in 5% CO_2_ atmosphere [21].

Data of patients with negative culture results were recorded and their stored CSF samples were further examined by real-time PCR.

### 2.1. Real-Time PCR

#### 2.1.1. Bacterial DNA Extraction

Bacterial DNA was extracted from CSF samples with the aid of QIAGEN DNA Mini kit (QIAGEN Inc., California) as per the manufacturer's protocol for DNA purification. The eluted DNA was stored at −20°C until further processed.

#### 2.1.2. Real-Time PCR with SYBR Green I

**Figure 1:**
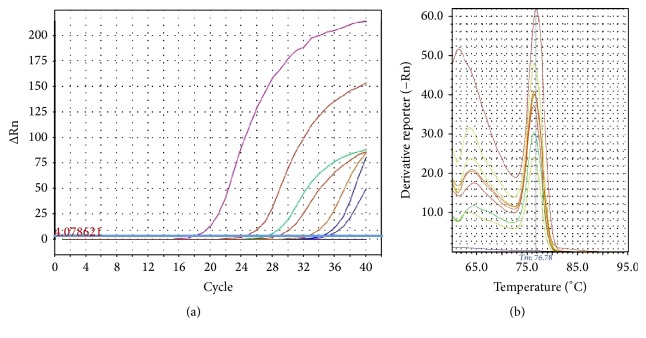
(a) Real-time PCR amplification plot for* lytA* gene specific for* Streptococcus pneumoniae*. Horizontal blue line represents the threshold value of fluorescence. (b) Melting curves of positive samples for* Streptococcus pneumoniae*.

Three runs were sequentially performed for the detection of each organism separately. The* ctrA* gene of* Neisseria meningitidis*,* bexA* gene of* Haemophilus influenzae*, and* lytA* gene of* Streptococcus pneumoniae* [9] were used as species-specific targets. The primers sequences are listed in Table 1. The mix for each run included 5 *μ*L of sample DNA, 12.5 *μ*L of 2x QuantiTect SYBR Green PCR master mix (QIAGEN Inc., Valencia, CA) containing a buffer, dNTP mix, MgCl_2_ and HotStarTaq DNA polymerase, 1 *μ*L of the primer, and RNase-free water for a final volume of 25 *μ*L. DNA was amplified with the Step One Real-Time PCR system (Applied Biosystems) by using the following temperature program: an initial Hotstart Taq activation step at 95°C for 15 min, initial denaturing at 95°C for 15 seconds and 40 PCR cycles of denaturing at 95°C for 30 seconds, annealing at 50°C for 30 seconds, and extension at 72°C for 30 seconds followed by melting curve stage of 95°C and 60°C [22]. Amplification data were analyzed by instrument software (Step One Software v2.3) in terms of melting curve graphs of each sample (Figure 1). Positive control strains and negative controls consisting of PCR grade water instead of the target DNA were used in each run.

### 2.2. Statistical Analysis

Quantitative variables are presented as mean and SD, and intergroup differences are compared using the unpaired *t*-test. Categorical variables are presented as number and percentage. Discrete and skewed continuous data are presented as median and differences between groups are compared using the Mann-Whitney test. The statistical procedures were carried out using SPSS version 15 for Windows (SPSS Inc., Chicago, IL, USA).

## 3. Results

The present study was conducted on 40 culture-negative CSF samples, withdrawn from 27 (67.5%) males and 13 (32.5%) females. Age of patients ranged from 19 to 56 years (mean ± SD = 38.33 ± 10.44). Demographic data of the patients and biochemical and cytological findings of the CSF samples are listed in Table 2.

CSF chemistry revealed elevated protein level (>50 mg/dL) in 82.5% of the samples and decreased glucose level (<40 mg/dL) in 87.5% of them. White blood cell (WBC) count >1.000 cells/mm^3^ was found in 57.5% of the samples whereas 42.5% of the samples showed WBC count between 100 and 1.000 cells/mm^3^. Predominant neutrophilic CSF (neutrophil > 50%) was found in 72.5% of the samples.

Thirty-six samples (90%) were positive for* Streptococcus pneumoniae* by real-time PCR, whereas* Neisseria meningitidis* and* Haemophilus influenzae* were not detected in any of the samples (0%). Four samples (10%) were negative for all three organisms (Table 3).

No statistical significant difference was observed regarding CSF chemistry and cells between samples of CSF positive and negative real-time PCR results (Table 4).

## 4. Discussion

Rapid and accurate laboratory diagnostics remain a crucial step for the final diagnosis of bacterial meningitis, specifying the treatment and implementing preventive measures for close contacts when indicated. Conventional culture methods, though the gold standard diagnostic technique, cannot be relied upon per se, in certain situations owing to the delay in results availability and the relatively limited sensitivity that had been repeatedly reported worldwide [12, 13, 23] and in conditions when prior antimicrobial therapy has been received. In Egypt, a high percentage of culture-negative samples was reported previously [24, 25]. These results were explained by the fact that most patients received antimicrobial agents that are readily purchased as over-the-counter medications even prior to clinical evaluation alongside the occasional delay in csf sampling. For the above-mentioned reasons, we employed a molecular method (real-time PCR) in this study to improve the diagnosis of bacterial meningitis in culture-negative purulent CSF samples.

Demographic data analysis of patients revealed that they were mainly males (67.5% versus 12.5% for females). Most of the cases belonged to the middle age group (mean = 38.3). This finding agreed with Fouad et al. [4] who confirmed that males were more significantly affected with bacterial meningitis than females (61% versus 39%, resp.) though the disease was distributed in all age groups, with low rates of occurrence in the extremes of age (the neonates and above 60 years).

Real-time PCR in this study was positive for* Streptococcus pneumoniae* in 36 culture-negative CSF samples (90%) while no positive results for* Haemophilus influenzae* or* Neisseria meningitidis* were detected. The age group of the patients in this study might have contributed to these results as the main causative agents of bacterial meningitis in adults are generally believed to be* Streptococcus pneumoniae* and* Neisseria meningitidis* [1, 26, 27].* Streptococcus pneumoniae* is found to be the commonest etiology of bacterial meningitis in the United States and Europe accounting for 61% of total cases in the United States [1, 3, 28] and in most African countries with high human immune deficiency virus prevalence [29, 30]. Yet meningococcal meningitis is common in Sub-Saharan Africa (the meningitis belt) but mostly apparent in the form of epidemics and outbreaks [31]. In a laboratory-based surveillance study undergone in Egypt by Afifi et al. [32], PCR was performed on purulent, culture-negative CSF specimens withdrawn from patients who met the criteria for case definition of bacterial meningitis.* Streptococcus pneumoniae* was also reported as the most common etiology of bacterial meningitis.

Fouad et al. [4] also documented* Streptococcus pneumoniae* as the most frequent isolate (52%) among bacteria causing meningitis. The agreement between our findings and those of the previously mentioned studies in Egypt consolidates the deduction of Shaban and Siam [33] in their review article that pneumococcal meningitis is currently the leading cause of meningitis in Egypt as its incidence is constantly rising at the expense of meningococcal meningitis, which may be a reflection of the increased use of polysaccharide meningococcal vaccines.

Wang et al. [7] also identified bacterial meningitis in five cases (9%) by CSF cultures and 25 (45%) by real-time PCR. They considered real-time PCR much more sensitive than culture for the diagnosis of bacterial meningitis particularly in their study where 68% of patients had received prior antimicrobial treatment and their CSF samples yielded negative culture results. A similar conclusion has been reached by Wu et al. [9] and Sacchi et al. [19] who stated that real-time PCR increases diagnostic yield for bacterial meningitis and is ideal for incorporation into routine surveillance in developing countries.

According to Brouwer et al. [34] CSF culture is documented to be positive only in 1/10th of the previously antibiotic treated patients in developing countries. Same result was found by Afifi et al. [32], who reported low rates of culture positive CSF samples (8%) of suspected cases with bacterial meningitis [32]. This low yield in culture results could be attributed to the fact that antimicrobials are being dispensed without prescriptions in Egypt.

This study faces the limitation of the relatively low number of CSF samples investigated and the lack of testing for other less commonly bacterial etiologies of adult meningitis (e.g., Group B* Streptococcus*,* Listeria monocytogenes*). This may provide an explanation for the negative results (10%) obtained.

According to our findings, we conclude that the use of molecular technique in the diagnosis of bacterial meningitis should be considered in suspected cases with negative culture results before reporting exclusion of the disease.

## Figures and Tables

**Table 1:** Real-time PCR primers [9].

Oligonucleotide	Sequence	Final conc. (nM)
*ctrA* forward	5′-TGTGTTCCGCTATACGCCATT-3′	300
*ctrA* reverse	5′-GCCATATTCACACGATATACC-3′	900
*bexA* forward	5′-TGCGGTAGTGTTAGAAAATGGTATTATG-3′	600
*bexA* reverse	5′-GGACAAACATCACAAGCGGTTA-3′	600
*lytA* forward	5′-ACGCAATCTAGCAGATGAAGCA-3′	200
*lytA* reverse	5′-TCGTGCGTTTTAATTCCAGCT	200

**Table 2:** Demographic, biochemical, and cytological data of patients (*n* = 40).

Characteristics	
*Age (years) *	38.33 ± 10.44

*Sex*	
Male	27 (67.5%)
Female	13 (32.5%)

*Protein (mg/dL)*	402.7 ± 346.84
<50	7 (17.5%)
>50	33 (82.5%)

*Glucose (mg/dL)*	23 ± 15.92
<40	35 (87.5%)
>40	5 (12.5%)

*WBCs (total/mm* ^*3*^)	7900.75 ± 12755.2
<100	0 (0%)
>100–1000	17 (42.5%)
>1000	23 (57.5%)

*Neutrophil percentage*	74.25 ± 20.87
<50%	11 (27.5%)
>50%	19 (72.5%)

Data are presented as mean ± standard deviation for continuous variables and as number (percentage) for categorical variables.

WBCs, white blood cells.

**Table 3:** Results of real-time PCR (*n* = 40).

PCR	Number	%
*Negative*	4	10%
*Positive *	36	90%
*Streptococcus pneumoniae*	36	90%
*Haemophilus influenzae*	0	0
*Neisseria meningitidis*	0	0

**Table 4:** Comparison between real-time PCR positive and negative samples as regards CSF cells and chemistry results.

Variables	RT-PCR positive	RT-PCR negative	*Z*/^*∗*^ *t*	*p *value
	Median			

Protein (mg/dL)	300	102.5	−1.873	0.061 (NS)
Glucose (mg/dL)	17	21.5	−0.339	0.735 (NS)
WBCs (total/mm^3^)	2215	400	−1.579	0.114 (NS)

	Mean ± SD			

Neutrophil percentage	75 ± 21.8	67.5 ± 18.5	^*∗*^0.66	0.513 (NS)

Data are presented as median or mean ± standard deviation.

NS: nonsignificant result (*p* value > 0.05).

*Z*: Mann-Whitney test.

^*∗*^ *t*: unpaired *t*-test.

WBCs, white blood cells.
